# Chatbots in Cancer Applications, Advantages and Disadvantages: All that Glitters Is Not Gold

**DOI:** 10.3390/jpm14080877

**Published:** 2024-08-19

**Authors:** Georgios Goumas, Theodoros I. Dardavesis, Konstantinos Syrigos, Nikolaos Syrigos, Effie Simou

**Affiliations:** 1 Department of Public Health Policy, School of Public Health, University of West Attica, 115 21 Athens, Greece; esimou@uniwa.gr; 2 Laboratory of Hygiene, Social & Preventive Medicine and Medical Statistics, School of Medicine, Faculty of Health Sciences, Aristotle University of Thessaloniki, 541 24 Thessaloniki, Greece; dardaves@auth.gr; 3 Oncology Unit, 3rd Department of Medicine, “Sotiria” Hospital for Diseases of the Chest, National and Kapodistrian University of Athens, 115 27 Athens, Greece; ksyrigos@med.uoa.gr (K.S.); nikolaos_syrigos@dfci.harvard.edu (N.S.); 4 Dana-Farber Cancer Institute, Boston, MA 02215, USA

**Keywords:** chatbots, advantages, disadvantages, challenges, artificial, intelligence, cancer, medicine, health

## Abstract

The emergence of digitalization and artificial intelligence has had a profound impact on society, especially in the field of medicine. Digital health is now a reality, with an increasing number of people using chatbots for prognostic or diagnostic purposes, therapeutic planning, and monitoring, as well as for nutritional and mental health support. Initially designed for various purposes, chatbots have demonstrated significant advantages in the medical field, as indicated by multiple sources. However, there are conflicting views in the current literature, with some sources highlighting their drawbacks and limitations, particularly in their use in oncology. This state-of-the-art review article seeks to present both the benefits and the drawbacks of chatbots in the context of medicine and cancer, while also addressing the challenges in their implementation, offering expert insights on the subject.

## 1. Introduction

In the 21st century, healthcare has undergone significant modifications due to the increasing number of patients with chronic medical conditions, the breakdown of the traditional hierarchy in medicine, easier access to new technologies, medical knowledge, and peer support online. This shift in culture, known as “digital health”, is becoming more evident. The roles of both patients and healthcare providers have also changed. Patients are now taking on a more proactive, empowered role and want to be active participants in their care. These “empowered patients”, also known as electronic patients (e-patients), are knowledgeable about managing their health or diseases, have access to information and technologies, and use electronic gadgets to collect data. Similar to this, the doctors’ roles are evolving into the “empowered physician or electronic physician (e-physician) who helps patients navigate the maze of digital information rather than acting as the gatekeepers to the ivory tower of medicine” [1].

Digital technologies, such as big data, the Internet of Things, virtual and augmented reality, smartphone and other apps, artificial intelligence (AI), social media, and chatbots, use synergy of computing platforms, connectivity, software, and sensors for healthcare and related uses, and they are currently constantly being applied to the medical and health fields, and have given new vitality to the evolution of what is called medical health [2]. Particularly, after the so-called coronavirus disease 2019 (COVID-19) pandemic, digital health has seen an astounding transformation [1,3]. The most significant change in this transition, though, is how digital health technologies put the patient at the center of care, enabling them to receive medical care or diagnosis wherever they are, whereas now, they take a more active role in their care, thanks to health monitors and mobile diagnostic gadgets that measure blood pressure, electrocardiography, fitness activities, and sleep quality, giving them access to data that previously were exclusively in the doctor’s files [1]. The ability to have natural language interactions with users through a variety of communication channels has made chatbots an indispensable component of our everyday life, and undeniably, the promotion of health and wellbeing could be greatly aided by such technology, yet various concerns have arisen towards the diagnosis of critical pathologies, such as cancer [4].

According to WHO declarations, cancer cases are projected to increase by 77% in 2050, with an estimated 35 million people having a history of cancer. Research data from 115 countries have shown that the majority of populations worldwide do not adequately prioritize cancer and palliative care services as a part of global health coverage. Only 39% of them cover basic cancer management, including pain management at a broader level, highlighting disparities in cancer care worldwide [5]. The International Agency for Research on Cancer (IARC) estimates that ten cancer types comprise about two-thirds of new cases and deaths, with lung, breast, and colorectal cancer being the three major types of cancer at a global level in 2022 [5]. However, receiving a cancer diagnosis seems to be much harder than the cancer itself. In this state-of-the-art review, the types, advantages, disadvantages, and challenges of novel chatbots in medicine, particularly in oncology, are thoroughly illustrated. Furthermore, expert opinions on the topic are thoroughly discussed.

In this novel and state-of-the-art review, the types, the advantages and disadvantages, and the challenges of the novel chatbots in medicine and particularly in oncology are thoroughly illustrated; furthermore, an expert opinion about this topic is thoroughly discussed. This original review will contribute to better understanding the advantages and highlighting the disadvantages of these chatbots and reveal their challenges and obstacles for medicine and cancer.

## 2. The Novel Chatbots in Medicine and Cancer

A chatbot, previously known as chatterbot, is a software program or web interface that simulates human conversations through text or voice interactions. Modern chatbots operate online and use advanced artificial intelligence technologies to converse in a natural language with users, mimicking human conversation patterns. While basic chatbots have existed for decades, contemporary versions often incorporate natural language processing and deep learning [6]. Scripted or rule-based chatbots typically rely on standardized user input through menus, tiles, or carousels, with responses based on predetermined rules. These types of chatbots are commonly used in marketing, customer service, and telecommunications to assist clients with common queries using pre-written scripts. Scripted chatbots do not support open-ended conversations. In contrast, AI chatbots, also known as AI conversational agents, process a natural language using neural networks or algorithms instead of relying on pre-written scripts [7]. However, chatbots can sometimes generate coherent yet incorrect or fabricated information, a phenomenon referred to as “hallucinations”. This occurs because chatbots predict responses rather than truly understanding the meaning of the input. When humans use and rely on chatbot-generated content tainted with delusions, it is referred to as “botshit” [8].

In 1950, Alan Turing published the influential article “Computing Machinery and Intelligence”, in which he introduced the Turing test as a measure of machine intelligence. This test evaluates a computer program’s ability to mimic human conversation in real time, making it difficult for a human judge to distinguish between the program and an actual human based solely on the conversation’s content [9]. Joseph Weizenbaum’s 1966 program “ELIZA” attracted attention, as it seemed capable of fooling users into believing that they were interacting with a real human. However, Weizenbaum did not claim that ELIZA was genuinely intelligent. Instead, he presented it as a debunking exercise [10]. Subsequent advancements included the addition of a paranoid patient’s personality to Kenneth Colby’s “PARRY” chatbot. One of the most well-known chatbots is “ALICE”, developed by Richard Wallace in 1995. ALICE avoids improper responses by using pattern-matching algorithms to retrieve example sentences from predefined output templates [11]. With the resurgence of interest in AI and machine learning, chatbots are increasingly prevalent and are being used in various domains. Recognizable web-based voice-activated assistants such as Microsoft Cortana, Apple Siri, Amazon Alexa, and Google Assistant have become widespread following the popularity of chat apps [12]. Despite these advancements, conversational artificial intelligence still faces broadscale challenges, and some software developers focus on using chatbots for information retrieval. Nowadays, chatbots are widely used in messaging apps, internal company platforms, politics, toys, data security, education, and healthcare [4,13,14,15,16,17,18].

A systematic review reported that, in 2021, there existed 5163 chatbots in the Botlist.co directory, out of which 95 were used for healthcare applications, such as diagnosis (12 chatbots), treatment, monitoring, support, workflow, and health promotion, and Messenger was the first reported chatbot in all these categories [11]. Actually, the initial medical chatbots were created and used to provide few easy automated responses to some common patient questions, including office hours and medication refill requests, as well as some other facilitating tasks, such as appointment scheduling. Chatbots were integrated into healthcare information websites via platforms like WebMD and marked an early stage in which they were determined to provide answers to user queries, and further developments include their integration into electronic health record systems so as to streamline administrative tasks and boost healthcare professional efficacy [19,20,21]. Recent developments, however, have thrust chatbots into crucial positions in relation to patient interaction and emotional counseling services, and of particular note, chatbots like Woebot have become important resources for mental health, facilitating meaningful dialogues and providing interventions based on cognitive behavioral therapy. This development highlights the groundbreaking ability of chatbots, including more recent versions like ChatGPT, to go beyond their original function of information delivery and actively engage in patient care; the ability of these AI-powered conversational bots to positively impact patient lifestyle and conduct decisions is becoming more and more apparent as they develop, changing the way that healthcare is delivered and how patients are treated [19]. These days, dozens of chatbots focused on fitness and health are accessible on the internet, and despite the fact that fitness bots are the most common in this area, there are also many medical bots that are valuable. Even if they are not yet widely used in healthcare, chatbots are becoming more and more popular; “FitCircle” and “GymBot” for fitness purposes, “Forksy” and “SlimMe” for nutrition guidance, and “Mendel Health” for oncology are a few prominent instances of specialized industry chatbots [22,23,24,25]. However, there are other issues with putting these technologies into practice and providing them, like the gradual changes in laws and the challenges of acquiring, processing, and retaining private information. Currently, diagnosing a patient in Russia requires a face-to-face consultation between the physician and the patient. Meanwhile, the Ministry of Medicine has mandated that half of all medical consultations must happen online by 2030 [26].

Actually, the so-called COVID-19 aided in pausing face-to-face consultation between the doctor and the patient; thus, it helped in telemedicine and online medical diagnoses and recommendations [27]. ChatGPT, the GPT chatbot, can respond to inquiries from users about disease prevention and health promotion, including immunization and screening, whereas WhatsApp and the WHO have partnered to create a chatbot service that responds to inquiries from users regarding COVID-19 [28]. Indeed, the Indian government introduced the “MyGov Corona Helpdesk” in 2020, which functioned via WhatsApp and provided users with information regarding the COVID-19 pandemic [29].

Chatbots’ architecture is based on a user interface via which the user interacts with it, the natural language understanding component that is responsible for accepting user inputs and extracting the appropriate information, the dialogue management that monitors the conservation’s flow and determines suitable responses, the integration layer that links it to external systems and databases to retrieve data or to act, and the analytics and feedback that monitor usage metrics and collect feedback for its improvement [30,31].

Currently, there exist five main types of chatbots in the domain of health. The data used to train the chatbot or provide readily available knowledge serve as the basis for classifying knowledge domains, including the closed domain, which focuses on more specialized material, and the open domain, which covers general issues. The classification of services is based on the user’s sentimental closeness to the provider and the degree of personal contact based on the task completed, and this can be further subdivided into intrapersonal and interpersonal domains for companionship and personal support to humans, information transmission services, and interagent conversation with other chatbots. The following classification is task-oriented, conversational, and informational, and it is based on goals that are intended to be achieved. Additionally, chatbots that generate responses are categorized as rule-based, retrieval-based, or generative, and they handle the task of interpreting inputs and producing outputs. Lastly, human computation is used in human-aided categorization, which offers greater robustness and flexibility but is slower to process more requests [32,33]. These types of chatbots and their certain recommended apps for each type of chatbots are presented in Table 1.

Particularly in the field of oncology, there exist certain chatbots with various purposes, from the examination of radiologic data to aid in clinical diagnosis and the monitoring of the symptomatology and seriousness of the disease and its stage, to the collection of family history so as to identify hereditary cancer cases, the examination of all data to generate treatment plans for oncologists, the entrance to care institutions and educational data, the daily mental support and tracking, and the general lifestyle coaching, healthy diet, and smoking cessation. Table 2 illustrates the commonly used chatbots with some examples of current certain applications of proposed designs [11].

## 3. The Benefits of Chatbots in Medicine and Cancer

From a general point of view, chatbots have multifarious advantages, with the most important being that they can provide support 24/7 at any time with instant response regardless of space, and also they provide answers automatically, thus achieving heightened lifetime value, robust brand affinity, and higher levels of satisfaction [34]. Additionally, they reduce the volume of calls and chats overall, allowing support professionals to concentrate on the most important discussions, and in this way, they help in reducing daily stress and anxiety and coping with stressful situations, not to mention also that chatbots can deliver messages that are emotionally comforting and customized to the stressors that users share with them [35,36]. The goal of contemporary chatbots is to converse with people in a conversational manner, and they can offer support in a friendly and engaging manner, as well as answer inquiries and make product recommendations [37]. Another benefit is the fact that some chatbot systems support multilingual text and speech in a fraction of a second, and they are crucial especially in healthcare, where they can support changes in behavior and they can also act like assistants and help with routine tasks and activities in living environments [38,39]. It should be highlighted that chatbots enable new user touch points, and also they improve convenience—through their easy navigation—and deep learning, and they reduce service and support costs for the users [40]. Because they gather useful user information during conversations, such as preferences, browsing history, and activity, chatbots are data-driven tools, and this information can be utilized to customize marketing campaigns, enhance existing products, and make wise decisions with no human mistakes and other manmade errors. Importantly, they display visual content that is more descriptive and realistic, and they provide compliance and security for the user’s personal information [41,42,43,44].

Particularly in the field of medicine, except from the aforementioned, chatbots offer some extra advantages. Chatbots such as ChatGPT have been characterized as beneficial for physicians and researchers, as they can aid in saving time and resources, as well as in providing fresh research perspectives [45]. Regarding the possible patients—the actual users—there are various more specific benefits, including 24/7 availability with rapid and daily responses for critical cases and for individuals with chronic medical conditions, standardized collection of data that can create an electronic health record, quick entry to important information for nearby hospitals/centers/pharmacies and operation hours, determination of assistance in case of a critical health condition requiring immediate attention and care, personalized and nuanced medication planning, scheduling of appointments integrated with online calendars and automatic reminders of them, diagnoses of rare or tricky illnesses and interpretation of complex diagnostic tests, and a lot of support for the person’s overall health, from physical activity to behavioral and mental health needs [46]. Moreover, in this way, some more efficacious patient self-services can be seen, since chatbots can help users to reach the appropriate data and stay healthy and safe, and they can provide a second and a third opinion on a diagnosis or medication plan, regardless of the user’s location [47]. Additionally, given that there are several divergent patient types, such as youngsters, elders, those with problems with their senses, those with mental or/and behavioral issues, those who speak English or not, and those coming from other countries, many chatbots provide a better patient engagement [48]. Aside from their better efficiency (compared with doctors), they can also scale more easily as most of the doctor’s time is spent speaking with patients over the phone or in person, but availability and geography restrict these connections. However, chatbots enable an unlimited number of people with an internet connection to request assistance whenever and from wherever, provided that someone is available to answer. Thus, physicians can more easily control patient demand without incurring additional fees, thanks to this scalability, and they can also maintain low costs while providing top-notch customer service [49]. Some other chatbot benefits in healthcare are the fast and personal interactions with fewer errors, real-time assistance, reduction in administrative tasks for health facilities, and elimination of a patient’s waiting [50].

Especially in cancer cases, conversational AI chatbots have been found to have the potential to provide patients with answers to their questions that are just as good, empathetic, and readable as those given by doctors, and expert AI chatbots that have been trained on large medical text corpora may provide vulnerable populations with information and act as point-of-care electronic medical tools. Additionally, they may offer emotional support to cancer patients and enhance oncology care [51]. Another study has shown that the most reported chatbot applications were for cancer screening, prevention, risk stratification, treatment, monitoring, and management, and the authors concluded that chatbots utilized in oncological care have so far shown a high degree of user satisfaction; several have been effective in enhancing patient-centered communication, making cancer-related information more accessible and facilitating access to care. At the moment, the main drawback of chatbots is their requirement for rigorous user testing and iterative development prior to broad deployment [52]. Other literature data from women living with breast cancer show that, in comparison with the “one size fits all” approach used by healthcare workers to provide information, chatbots are practical and affordable tools to help improve and increase self-care practices and reduce the side effects of chemotherapy, and they can also act as empowering tools to support nurses in educating women with breast cancer and empower them to take an active role in dealing with their symptoms [53]. Importantly, a study on women with breast cancer found that the chatbot’s EORTC INFO25 scores were not less than the doctors’ scores in providing the answers the women needed [54].

## 4. The Disadvantages of Chatbots in Medicine and Cancer

Some major disadvantages of chatbots are their dependence on technology, internet connection, and the overall automation in general; since chatbots depend on an internet connection to work, users in places with spotty or inconsistent internet connectivity may find it difficult to use chatbot services, yet it is important to take into account the user base’s potential restrictions, particularly with regard to patients [55]. Of course, chatbots have to adapt to the new technology as it evolves and expectations change. Despite the fact that these AI technologies reduce customer costs, they have high initial development costs and maintenance costs [56]. Additionally, using chatbots means that the user has relevant knowledge about this technology; not only are these chatbots complex in certain cases—especially for patients whose condition determines the extent to which they can use them—but also they can cause frustration and aggression for some users because if AI misinterprets the user’s meaning or give irrelevant replies, users may become irritated—something that can also happen if the user cannot handle them appropriately [57]. As is typically the case with technologically driven modifications to current services, some customers—mostly those from older generations—find chatbots uncomfortable because of their limited comprehension, which makes it clear that the robots are handling their demands. Conversations with users that are non-linear and need back-and-forth exchanges are challenging for chatbots to handle [58]. The efficiency of a chatbot is mostly dependent on language processing, which is limited by anomalies like accents and errors, and additionally, because the input/output database is fixed and has a finite capacity, chatbots may malfunction when attempting to answer an unsaved query [59]. While generative neural networks, which are built on algorithms that use deep learning to produce novel answers word by word depending on user input, are often trained on an enormous amount of natural language phrases, chatbots require a large quantity of conversational information to train [60]. Furthermore, apart from the ethical and privacy questions raised about chatbots such as ChatGPT, using them to handle private or sensitive client data might lead to security issues, and it is essential to make sure that data are encrypted, stored safely, and shielded from unwanted access, because security lapses may result in serious repercussions, such as harm to one’s reputation and legal status [61,62].

Chatbots have some extra disadvantages in the medical field, even from a physician’s and a medical researcher’s point of view, since, even for them, the information that they provide maybe not accurate enough [33]. Importantly, adoption is hampered, among other things, by algorithmic and data access restrictions, laws, an excessive dependence on data, as well as a lack of time or resources to undertake research while simultaneously managing and caring for human lives; chatbots run a higher risk of producing erroneous or defective solutions since they require constant problem solving when using a network-generated algorithm [63,64]. Additionally, a lack of transparency causes healthcare professionals to use them less and have less faith in them, whereas adoption is further hampered by poor information availability, a lack of time for clinical research, and a laborious data collection procedure that may negatively affect patient flow [65,66]. Additionally, the inability to gather huge data sets hinders technology adoption, and the matter of delayed implementation is aggravated by the extremely tightly controlled structure of healthcare, risks associated with liability, privacy difficulties, and an onerous approval procedure for the latest innovations [63,64]. Moreover, based on their philosophy, no human interaction is always offered, and some people may feel shy and uncomfortable to talk with an automated non-human system and share personal information and very sensitive health matters, and chatbots may not be as trustworthy to some as a genuine person, who can give them tailored advice and respond to their questions instantly—particularly for cancer or other serious health problems [65]. Apart from the limited information, the security concerns, the system overload, and the issues regarding data inaccuracies and credibility, chatbots rely on big data and AI; by using a chatbot for healthcare services, this may indicate that numerous organizations have gained access to your private details, while big data and AI can be costly for startups or small businesses that do not currently have the resources to operate efficiently or still need access to this kind of technology [50,66]. Last but not least, it should be noted that, aside from the incapability of chatbots to give access to healthcare professional and specialists, it must be highlighted that, sometimes, they provide inaccurate medical advice, as, given that they are human-programmed, they are prone to mistakes, some of which can be extremely harmful and serious, such as providing the patient with incorrect medical instructions or implying that they have developed a new, nonexistent ailment. Such misdiagnosis can occur due to various reasons: erroneous system diagnosis, wrong estimation from AI, inadequacy of chatbot questions, and inaccurate data provided by the user either due to distress/uneasiness or underestimation/negligence of their symptomatology [67,68].

**Figure 1:**
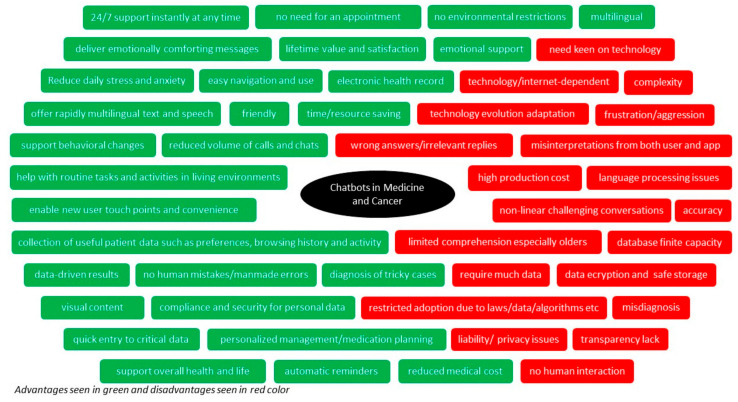
Advantages and disadvantages of chatbots in medicine and cancer.

Particularly in oncology, in one retrospective, cross-sectional study, the researchers assessed how well OpenAI’s commercial ChatGPT produced treatment recommendations for lung, prostate, and breast cancer, and compared those recommendations with the National Comprehensive Cancer Network standard of care, and they discovered that about one-third of the chatbot’s treatment recommendations deviated from the its guidelines, and the recommendations differed depending on how the questions were phrased. Differences between the guidelines and the chatbot’s output were frequently ascribed to unclear answers, suggesting that care should be used when utilizing chatbots to obtain treatment-related information [69]. In a relevant study, researchers contrasted the quality of data produced by ChatGPT v3.5 with that of Perplexity, Chatsonic, and Bing AI for the top Google search queries pertaining to five common cancers (lung, skin, colorectal, breast, and prostate), and despite having an excellent median DISCERN score, the replies were difficult to grasp and not easily actionable at the collegiate reading level [70]. Figure 1 shows all the advantages and disadvantages of chatbots in medicine and cancer, as previously discussed.

Based on these, the aforementioned advantages and disadvantages of chatbots in medicine and cancer seen in Figure 1, the following Table 3 presents the major implications of the chatbots for cancer.

Therefore, it is obvious that, regardless of the various advantages that the chatbots in cancer in particular may offer, there arise various disadvantages as well that should be taken into account before using them.

## 5. The Current Issues and Challenges of Chatbots

The major challenges of chatbots at first glance include the excellent design of its style, data gathering and algorithms, and the need for continuous maintenance; nevertheless, understanding the messages and figuring out the user’s intention is one of the main problems in using chatbots for customer service since, when creating a chatbot, the first thing that has to be done is to program adaptable algorithms for determining the message’s intention [71]. Personalization is another challenge of chatbots, yet the difficulty lies in figuring out the best ways to adjust to the user, and it can only be resolved by making several attempts and failing in each unique situation [71,72,73]. A significant obstacle associated with tailoring and modifying chatbot behavior is comprehending the limitations of natural language processing, since, while it is the foundation of every chatbot, the overuse of it may be compared with thinking up an angry, upside-down elephant in a cloud, whereas, put another way, it might turn out to be just as confusing as any periods of a cat sitting on the keyboard; however, that is an extreme example. Things are not always so bad, but miscommunication takes place [74]. Another option is the machine learning, but for it to work well, it requires a very specific set of guidelines, and if not, chaos will ensue. Moreover, undeniably, another major challenge for chatbots is digital literacy, because there exist various types of users, and especially old ones and those who are not able to use such technology—due to either the absence of knowledge or other personal or health reasons—may be not able to use a chatbot or/and interpret the meanings of its responses [75].

Particularly in the field of healthcare, to be able to identify specific patterns in big data, developers and suppliers of AI/machine learning-driven medical products need a significant amount, speed, variety, and veracity of medical data [76]. Additionally, it was previously discussed that privacy concerns are a common obstacle, along with the user’s capability of providing real answers and his/her trust—which is affected by various factors apart from empathy and emotional intelligence [77]. One thing that restricts the usage of chatbots is that, in the early phases of their creation or use, they might not be able to comprehend patients well enough to respond appropriately to certain types of questions. It has been said that chatbots in medicine are a medical supplement and not a substitute, because they remain short of the human qualities of empathy, instinct, and prior experience that make healthcare workers human [78]. Furthermore, AI chatbots raise serious questions about computational bias and fairness as they become more and more prevalent in the healthcare industry, as they may reflect the inherent biases or lack of diversity in the training data. It is crucial to make sure that the creation and application automated chatbot models within the healthcare industry follow the rules of justice and equity because of the possibility of unfavorable results, because reaching this goal can support fair healthcare outcomes and access for all populations, irrespective of their demographic makeup [79,80,81]. Moreover, AI chatbots in the healthcare industry may face substantial challenges navigating regulatory environments; besides, the fast developing field of chatbot software and the absence of industry standards in chatbot applications exacerbate the difficulties involved in regulatory assessment and supervision [82,83]. It should also be mentioned that, apart from acceptability, health literacy matters too; users should be capable of reading and comprehending the chatbot’s outputs, yet there are various users with different knowledge, and this is a next-stage limitation of digital health [84,85]. Last but not least, even if doctors currently use technology and AI to help them to find appropriate ways to deliver breaking bad news to a patient, chatbots are not humans, and it seems difficult for a non-human means to communicate bad news with the user—who is actually a human—particularly for diagnosis, but also for therapeutic strategies or other issues, not to mention that a possible false diagnosis has detrimental consequences to the user [86]. Despite their acceptance by doctors, there are still issues with chatbots’ incapacity to understand human emotion and situations when medical intelligence and expertise are needed [87].

**Figure 2:**
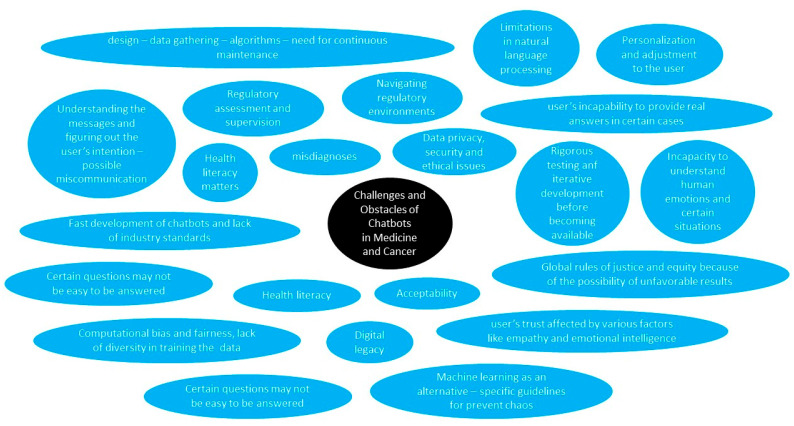
Current challenges and obstacles of chatbots in medicine and cancer.

It was discussed in the previous section that the main drawback of chatbots in oncology is that they must undergo rigorous user testing and iterative development before being widely used [52]. As with all diseases and other reasons to use a chatbot, challenges include data privacy, security, and ethical aspects [88]. Figure 2 summarizes the current challenges and obstacles of chatbots in medicine and cancer.

## 6. The Expert Opinion on Chatbots and Oncology: All That Glitters Is Not Gold

AI technology and novel chatbots have remarkably revolutionized modern life to a great extent. Given the fact that chatbots in oncology are not such common but will be in the near future, several points and aspects from this critical state-of-the-art review should be taken into account, so as for this technology to be widely accepted, adopted, and efficient for society. In the previous sections, the benefits, the disadvantages, and the challenges of novel chatbots have been thoroughly presented. In this section, some crucial aspects about this topic will be extensively illustrated.

Undoubtedly, chatbots offer several advantages for the user and society, but a lot of disadvantages and challenges are behind them. Nevertheless, some efforts to overcome these obstacles have been recommended, such as regularly updating the chatbot’s base of knowledge, incorporating machine learning and natural language processing, administering data validation and verification, ensuring data safety, and providing human assistance wherever possible [45]. Not only will chatbots be beneficial for researchers, academics, and even university medical students for education purposes, but also they will help in societal health literacy, and in this way, more and more people will have knowledge on health aspects; even physicians have characterized them as being undergraduates in the school of medicine and new members in the medical society [1].

Of course, prevention is better than cure, and an early diagnosis can show promising results; many chatbots aid in the prevention and the early diagnosis of communicative and non-communicative diseases—with the first being critical especially in cases of epidemics and pandemics such as COVID-19. However, if one is digitally illiterate and not familiar with AI technologies and chatbots, then he will not be diagnosed appropriately through them, and he will continue to be ill; this scenario is critical particularly for communicable diseases that have consequences at the societal level and serious conditions characterized by underlying systemic inflammation, such as autoimmunity and cancer [89]. Given the fact that there are a growing number of people with such underlying medical conditions, particularly elders who also may not be familiar with AI and chatbots, one could argue that this is a major obstacle and difficult to overcome.

Chatbots are personalized, but they may not be personable. Behind the digital scenes, there exists the same impersonality, and one’s history cannot be totally monitored through them; such complex algorithms would cause a crash into the system and trigger incorrect outputs to the user. Additionally, chatbots are capable of producing coherent-sounding but erroneous or invented information, a phenomenon known as “hallucinations”, because they predict answers rather than understanding the meaning of the responses [9]. This AI technology uses determined standard questions in order to have results, yet symptoms can overlap and occur due to other underlying medical conditions, and even differential diagnosis is evident in the output; the user—who is not an expert—cannot interpret it. Clinical judgment is not entirely replicated by AI systems; factors such as patient composure, cognitive function, and clinical state are important in determining patient danger but are not conclusively recorded in data. It is possible to increase model accuracy and beneficial relevance by extending prognostic AI models to incorporate patient-reported outcomes that continuously record symptoms and functional ability outside of the clinic. Importantly, a user can report fake symptomatology and trigger again a false diagnosis; however, fake results and misdiagnosis are possible not only in digital diagnosis but also in clinical and laboratory diagnosis—the last was recently seen in false COVID-19 test results and even Progressive Multifocal Leukoencephalopathy diagnosis [90,91,92,93,94,95,96].

Whether it be a real or false diagnosis, in most cases, breaking bad news are delivered to the possible patient. In case of an accurate diagnosis, one could argue that it is cold and inhumane for a patient to be informed about a poor prognosis, a bad or worsened diagnosis, or a failure in therapy via a chatbot. This would be important particularly in cancer cases, whose psychology is extremely affected by such diagnosis. In case of a false diagnosis, one could argue that this could have significant consequences on one’s mental health and his family, since a cancer diagnosis is believed to be a heavy burden and even a stigma for most people.

Communication and interactions between patients, caregivers, healthcare practitioners, and the larger healthcare ecosystem are all included in the diverse field of healthcare communication. It has long been understood that good communication is essential to providing high-quality healthcare, and it is essential for early health issue discovery, treatment plan adherence, patient education, and general patient happiness; however, traditional healthcare communications on strategies have faced both opportunities and challenges with the arrival of the digital age [19]. Of course, digital diagnosis cannot substitute clinical and laboratory diagnosis, and in case of prognosis, diagnosis, and therapeutic strategy planning, communication between patients and experts is the gold standard; this is a very important parameter that influences the patient’s outcomes—especially in cancer cases. Such people face some extra obstacles, particularly psychological ones, since psychology matters in cancer, and of course, the disadvantages, obstacles, and challenges of chatbots for cancer are much heavier—compared with other health conditions.

## 7. Conclusions

AI technology and novel chatbots have remarkably revolutionized modern life, particularly in the field of medicine. Despite the fact that such technologies have various advantages, there exist disadvantages too, and important challenges and obstacles have arisen, especially with regard to their use in cancer cases. Of course, people, and especially cancer cases, should be informed about the chatbot technology and use it only in a few specific and safe situations when the benefits outweigh the risks.

## Figures and Tables

**Table 1:** The five main types of chatbots on health and their certain recommended apps.

Knowledge domain	The open domain responds to broader categories that can be easily searched within databases, and it may be preferred in cases of routine screening of the symptomatology or connecting to providers/services or health-promoting apps. The closed domain responds to direct or difficult questions that require thorough research, and may be utilized for a treatment plan or recommendations.
Service-provided	The interpersonal services are mostly chosen to disseminate information in intimate connections and may be preferred in imaging diagnostics or hereditary evaluation whose purpose is to relay real information to users. The intrapersonal services are used for support and communication purposes, and they may be preferred for counseling, emotional support, and health promotion that requires some human touch. The interagent services are preferred for communication with other chatbots/computing systems, and they may be the preferred chatbot type for administration when transferring patient data among locations.
Goal-based	The informative type provides data from warehouse databases/inventory entry, and it may be chosen for linking patients with resources or the remote monitoring of patients. The conversational type’s purpose is to converse with users in a natural base, and it may be used in counseling, support, and the promotion of health. The task-based type is single-acting in predetermined cases, and it is preferred in screening and diagnostics.
Response generation	It uses certain algorithms in narrow domains, and efficient evidence is available to train the system, and it is again used for screening and diagnostics.
Human-aided	It incorporates human computation with increased flexibility and robustness but decreased speed, and it is preferred for most apps except for support and workflow efficiency that require speed so as to deliver care immediately.

**Table 2:** The cases of applying the commonly used chatbots in oncology.

Screening and diagnosis	Imaging diagnostic	Medical Sieve	Assesses radiologic data to help physicians’ diagnosis
	Symptom screening	Quro	Presynopsis relying on symptomatology and history to predict user’s situation
		Buoy Health	Aids in finding the etiology of diseases and provides medical recommendations
		Harshitha breast cancer screening	Dialog flow providing an early diagnosis of symptomatology of breast cancer
		Babylon	Symptom checker
		Healthily (formerly Your.md)	
		Ada	
	Hereditary assessment	ItRuns	Collects family history data at a populace level to assess hereditary cancer
Treatment	Patient treatment recommendation	Mathew	Detects symptomatology and predicts the illness via a symptom–illness data set and provides appropriate treatment
		Madhu	Offers a catalog of available therapies for different illnesses and provides data to the user for the synthesis and prescribed use of medications
	Connecting patients with providers or resources	Divya	Engages users to provide a personalized diagnosis and links to suitable healthcare
		Rarhi	Offers a symptom-based diagnosis and counts the seriousness and links with a physician
	Physician medication planning	Watson for Oncology	Evaluates information from medical records to provide a medication plan for physicians
Monitoring	Remote patient monitoring	STREAMD	Offers entry to healthcare instructions and educational data
		Conversa	
		Memora Health	
		AiCure	Coaches patients to control their condition and follow the instructions
		Infinity	Estimates outcomes and consequences of mobile-based monitoring for cancer cases aged ≥65 years
		Vik	informs about cases’ routine needs and concerns
Support	Counseling	Vivobot	Cognitive and behavioral psychological help
	Emotional support	Youper	Daily support and tracking mental condition
		Wysa	
		Replika	
		Unmind	
		Shim	
		Woebot	
Workflow efficiency	Administration	Sense.ly	Controls appointments and patients’ conditions and recommends medications
		CareSkore	Monitors vitals and informs the need for admissions to the hospital
		Mandy	Aids healthcare staff by automating case’s intake process
	patient encounter	HOLMeS	helps diagnosis, chooses the suitable medications and offers preventative check-ups
Health promotion	General lifestyle coaching	SWITCHes	Monitors case’s progress, offers help to physicians, and recommends appropriate activities
		CoachAI	
		WeightMentor	Offers self-help activation for maintaining body weight and an open dialogue
	Healthy eating	Healthy Hero	Aids in deciding for foods to alter unhealthy eating habits
		Tasteful Bot	Cognitive behavioral medication
		Forksy	
		SLOWbot	
	Smoking cessation	SMAG	
		Bella	Helps stopping smoking

**Table 3:** The major implications of the chatbots for cancer.

Medical Sieve	Advantages	Disadvantages
Quro	Automation, 24/7 availability, low operational costs, scalability, uniform responses, data processing, multilingual, remote access	Limited understanding in complex queries, contextual awareness, inaccuracies, required maintenance, data privacy, cybersecurity, biases, transparency, automation impact, empathy
Buoy Health	24/7 availability, remote access, symptom checking, tailored advice, time saving, resource optimization, informed choices, next steps guidance	Diagnostic limitations, algorithm dependence, privacy and security concerns, limited scope, over-reliance, technical issues, ethical and regulatory challenges like bias and fairness
Harshitha breast cancer screening	Early detection, increased awareness, self-examination, improved imaging techniques, risk reduction	False positives and false negatives, overdiagnosis of non-threatening cancers, impact on the overall quality of life, radiation exposure, financial burden, accessibility issues
Babylon	24/7 availability, remote access, reduced waiting times, quick symptom checking, affordable services, insurance integration, tailored advice, health monitoring, data-driven	Diagnostic limitations in complex cases, privacy and security issues, technology-dependent, app malfunctions, limited human touch, communication barriers, regulatory compliance, biases and fairness
Healthily (former Your.md)	24/7 availability, remote access, free services, reduced health costs, user-friendly interface, tailored advice, personal health records, data protection	Diagnostic limitations, algorithm dependence, limited scope, lack of human touch, communication barriers, internet-dependent, technical issues, over-reliance on self-diagnosis
Ada	24/7 availability, remote access, user-friendly interface, personalized health assessments, educational resources, privacy and security	Diagnostic limitations, algorithm-dependent, limited scope, lack of human touch, communication barriers, technology/internet-dependent, over-reliance on self-diagnosis
ItRuns	Early detection and prevention, tailored information, customized recommendations, advanced testing, broad coverage, awareness and understanding, supportive guidance, data security and privacy	Psychological impact, false positives and false negatives, complex interpretation, high costs, accessibility issues, privacy and ethical concerns, uncertain outcomes, scope limitations
Mathew	Early detection and prevention, tailored healthcare plans, risk reduction strategies, targeted therapies, detailed risk assessment, family health insights, informed decision-making, health literacy, at-home testing, online support	Psychological impact, false positives and false negatives, complex results, high costs, accessibility, data privacy, genetic discrimination, variable risk, incomplete coverage
Madhu	Early detection and prevention, tailored recommendations, custom health plans, informed decisions, health awareness, at-home testing, online results, data protection	Psychological impact, false positives and false negatives, misinterpretations, financial barriers, accessibility issues, data privacy, genetic discrimination, partial coverage, lifestyle and environmental factors
Divya	Proactive health management, customized preventive strategies, tailored recommendations, custom health plans, at-home testing, online results and support, informed decision-making, health literacy, data protection	Psychological impact, false positives and false negatives, misinterpretations, financial barriers, accessibility issues, data privacy, genetic discrimination, partial coverage, lifestyle and environmental factors
Rarhi	Proactive health management, preventive interventions, customized recommendations, tailored health plans, at-home testing kits, online results, understanding genetic risks, health awareness, data protection	Psychological impact, false positives and false negatives, complex results, financial barriers, limited access, data privacy, genetic discrimination, partial coverage, lifestyle and environmental factors
Watson for Oncology	Evidence-based recommendations, up-to-date information, rapid analysis, time saving, integration of multimodal data, personalized treatment, knowledge sharing, training and support	Performance variability, error rate, health system integration issues, data compatibility, data limitations, dependence on data quality, high costs, training and support, liability issues, patient privacy
STREAMD	comprehensive data view, improved data access, predictive analytics, personalized care, streamlined processes, clinical alerts, patient portal, health monitoring	data quality, false positives and false negatives, system compatibility, data standardization, data privacy, risk of breaches, high costs, training needs, technical issues, over-reliance on AI
Conversa	Improved communication, patient education, remote access, 24/7 availability, tailored interactions, health monitoring, streamlined processes, data integration, actionable insights, performance tracking	System compatibility, technical issues, data privacy, regulatory compliance, implementation costs, subscription fees, learning curve, resistance to change, reduced face-to-face interactions, possible miscommunication
Memora Health	Personalized communication, improved adherence, 24/7 availability, remote monitoring, automated workflows, data integration, centralized communication, collaborative tools, actionable analytics, performance tracking	System compatibility, technical issues, data privacy, regulatory compliance, implementation costs, subscription fees, user adoption and training–learning curve and resistance to change, over-reliance on automation, miscommunication risks
AiCure	Real-time monitoring, adherence support, interactive interface, behavioral insights, data-driven outcomes, automated process, remote management, improved data accuracy, real-time feedback	Data privacy, compliance issues, system compatibility, technical issues, user adoption and training–learning curve and resistance to technology, AI limitations, false positives and false negatives, implementation costs, subscription fees
Infinity	Comprehensive data integration, streamlined processes, tailored recommendations, adaptive algorithms, data-driven insights, predictive analytics, remote access, 24/7 availability, enhanced data collection, real-time monitoring	Data privacy, regulatory compliance, system compatibility, technical glitches, high costs, subscription fees, user adoption and training–learning curve and resistance to change, dependence on data quality, possible misinterpretations
Vik	Personalized recommendations, 24/7 accessibility, automated processes, streamlined workflows, actionable analytics, predictive capabilities, remote access, user-friendly interface	Data protection, compliance, system compatibility, technical problems, high implementation costs, subscription fees, training needs, resistance to change, dependence on data quality, possible misinterpretations
Vivobot	AI-powered conversations, behavioral insights, medication reminders, health monitoring, streamlined engagement, data collection, accessible everywhere, real-time feedback	Data security, regulatory compliance, AI limitations, technical issues, implementation costs, ongoing fees, user adoption and training–learning curve, resistance to new technology, dependence on data accuracy, miscommunication risks
Youper	Tailored interactions, adaptive learning, 24/7 availability, easy to use, mood monitoring, data insights, real-time feedback	Limited understanding, miscommunication risks, data privacy and security, compliance issues, resistance to technology, technology-dependent, subscription fees
Wysa	Evidence-based cognitive behavioral therapy and therapeutic exercises, 24/7 accessibility, user-friendly, custom interactions, mood tracking, connection to human therapists	Lack of human empathy, misunderstanding risks, sensitive information, compliance issues, high cost for premium features, technology-dependent
Replika	Companionship, conversational AI, self-improvement tools, emotional insights, 24/7 availability, easy to use, personalized experience,	Limited emotional depth, miscommunication risks, data privacy and security, regulatory compliance, AI-dependent, in-app purchases
Unmind	Holistic approach, evidence-based content, tailored for organizations, engagement metrics, 24/7 accessibility, user-friendly, proactive approach	Sensitive data, compliancy, privacy and security concerns, genetic resources, employee participation, subscription fees
Shim	Customized interactions, behavioral insights, 24/7 availability, user-friendly, immediate support, adaptability	Complex issues misinterpretation, miscommunication risk, sensitive data handling, compliance, privacy and security concerns, user engagements, potential costs for premium features/services
Woebot	Evidence-based cognitive behavioral therapy, research backed, 24/7 support, user-friendly, real-time conversations, affordable access	Limited depth, miscommunication risks, data privacy and security, regulatory compliance, resistant to technology, dependency risk
Sense.ly	Virtual assistant, real-time interaction, patient tracking, data integration, accessible anywhere, 24/7 availability, personalized interactions	AΙ limitations in understanding complex conditions, possible miscommunications, data privacy and security, regulatory compliance, system compatibility, technical issues, implementation costs
CareSkore	Risk evaluation, personalized recommendations, holistic view, continuous monitoring, intuitive design, accessibility, early intervention	Sensitive data handling, regulatory compliance, dependence on data quality, misinterpretation risks, subscription fees, adherence challenges
Mandy	Tailored resources, adaptive learning, 24/7 access, user-friendly, variety of resources, behavioral insights, data protection	AI limitations in understanding complex issues, miscommunication risks, user motivation, cost consideration for some premium features, technology-dependent
HOLMeS	Personalized interventions, health tracking, comprehensive health data, real-time updates, interactive tools, educational content, multi-platform availability	Sensitive information, regulatory compliance, user learning curve, integration challenges, complexity, implementation costs, AI algorithm accuracy
SWITCHes	Personalized coaching, goal setting, interactive features, feedback mechanisms, 24/7 availability, user-friendly, evidence-based scientific principles	Privacy and security, data management, regulatory compliance, user adherence, subscription fees, personalization AI limits
CoachAI	Tailored recommendations, adaptive learning, 24/7 accessibility, user-friendly, data-driven insights, real-time feedback, scalability	Understanding complex needs, miscommunication risks, data privacy and security, regulatory compliance, motivation challenges, subscription costs, usage fees
WeightMentor	Customized plans, adaptive feedback, comprehensive tracking, data insights, motivation tools, educational content, ease to use	Complex AI needs, misinterpretation risks, sensitive data handling, regulatory compliance, consistency, subscription fees
Healthy Hero	Comprehensive wellness, personalized recommendations, informative content, interactive features, goal setting, progress insights, motivation tools	Personalization limits, possible miscommunication, data privacy and security, regulatory compliance, consistency, cost for premium features
Tasteful Bot	Nutritional guidance with healthy recipes and nutritional information, dietary preferences based on user and adaptive suggestions, easy access, 24/7 availability, interactive experience	Limitations in complex dietary needs, risks for inaccurate recommendations, data management, regulatory compliance, adherence challenges, costs for premium features
Forksy	Tailored recommendations, adaptive algorithms, comprehensive health view, real-time monitoring interactive features, educational content, accessible anywhere	Limitations in complex conditions, data accuracy, data privacy and security, regulatory compliance, consistency, potential fees
SLOWbot	Slow-paced interactions, stress reduction, engaging experience, personalization, accessible anytime, easy to use, mindfulness resources,	Depth of support, possible miscommunication, user adherence, data management, niche focus
SMAG	Real-time tracking, adaptive recommendations, holistic view, actionable insights, interactive tools, educational resources, accessible across devices	Limitations in accuracy and interpretation, complex health issues, sensitive data, data privacy and security issues, regulatory compliance, subscription fees, adherence
Bella	Comprehensive approach, personalized interactions, engaging experience, real-time feedback, informative content, 24/7 access, user-friendly	Depth of support, miscommunication risks, data privacy and security issues, consistency, cost for premium features

## References

[1] Meskó B. (2022). COVID-19’s Impact on Digital Health Adoption: The Growing Gap between a Technological and a Cultural Transformation. JMIR Hum. Factors.

[2] Yang K., Hu Y., Qi H. (2022). Digital Health Literacy: Bibliometric Analysis. J. Med. Internet Res..

[3] Mouliou D.S., Pantazopoulos I., Gourgoulianis K.I. (2022). COVID-19 Smart Diagnosis in the Emergency Department: All-in in Practice. Expert. Rev. Respir. Med..

[4] Xue J., Zhang B., Zhao Y., Zhang Q., Zheng C., Jiang J., Li H., Liu N., Li Z., Fu W. (2023). Evaluation of the Current State of Chatbots for Digital Health: Scoping Review. J. Med. Internet Res..

[5] Global Cancer Burden Growing, amidst Mounting Need for Services. https://www.who.int/news/item/01-02-2024-global-cancer-burden-growing--amidst-mounting-need-for-services.

[6] McTear M., Callejas Z., Griol D. (2016). The Conversational Interface.

[7] Nicolescu L., Tudorache M.T. (2022). Human-Computer Interaction in Customer Service: The Experience with AI Chatbots—A Systematic Literature Review. Electronics.

[8] Hannigan T.R., McCarthy I.P., Spicer A. (2024). Beware of Botshit: How to Manage the Epistemic Risks of Generative Chatbots. Bus. Horiz..

[9] Turing A.M. (1950). I.—Computing machinery and intelligence. Mind.

[10] Weizenbaum J. (1966). ELIZA—A Computer Program for the Study of Natural Language Communication between Man and Machine. Commun. ACM.

[11] Xu L., Sanders L., Li K., Chow J.C.L. (2021). Chatbot for Health Care and Oncology Applications Using Artificial Intelligence and Machine Learning: Systematic Review. JMIR Cancer.

[12] AbuShawar B., Atwell E. (2015). ALICE Chatbot: Trials and Outputs. Comput. y Sist..

[13] Amalia A., Suprayogi M. (2019). Engaging Millennials on Using Chatbot Messenger for Eco-Tourism.

[14] Chandel S., Yuying Y., Yujie G., Razaque A., Yang G., Arai K., Kapoor S., Bhatia R. (2019). Chatbot: Efficient and Utility-Based Platform. Proceedings of the Intelligent Computing.

[15] Kim Y., Lee H. (2023). The Rise of Chatbots in Political Campaigns: The Effects of Conversational Agents on Voting Intention. Int. J. Hum. Comput. Interact..

[16] Lin P.-C., Yankson B., Lu Z., Hung P.C.K. Children Privacy Identification System in LINE Chatbot for Smart Toys. Proceedings of the 2019 IEEE 12th International Conference on Cloud Computing (CLOUD).

[17] Lai S.-T., Leu F.-Y., Lin J.-W., Barolli L., Leu F.-Y., Enokido T., Chen H.-C. (2019). A Banking Chatbot Security Control Procedure for Protecting User Data Security and Privacy. Proceedings of the Advances on Broadband and Wireless Computing, Communication and Applications.

[18] Liu L., Subbareddy R., Raghavendra C.G. (2022). AI Intelligence Chatbot to Improve Students Learning in the Higher Education Platform. J. Interconnect. Netw..

[19] Sun G., Zhou Y.-H. (2023). AI in Healthcare: Navigating Opportunities and Challenges in Digital Communication. Front. Digit. Health.

[20] Goel R., Goswami R.P., Totlani S., Arora P., Bansal R., Vij D. Machine learning based healthcare chatbot. Proceedings of the 2022 2nd International Conference on Advance Computing, Innovative Technologies in Engineering (ICACITE).

[21] Kocakoç I.D. (2022). The role of artificial intelligence in health care. The Impact of Artificial Intelligence on Governance, Economics, Finance.

[22] FitCircle: Where Bots Make Daily Eating Habits Healthier. https://www.firstpost.com/tech/startup/fitcircle-where-bots-make-daily-eating-habits-healthier-3725943.html.

[23] Sawers P. (2016). Gymbot: A Bot That Tracks Your Workouts through Facebook Messenger.

[24] Rahmanti A.R., Yang H.-C., Bintoro B.S., Nursetyo A.A., Muhtar M.S., Syed-Abdul S., Li Y.-C.J. (2022). SlimMe, a Chatbot with Artificial Empathy for Personal Weight Management: System Design and Finding. Front. Nutr..

[25] Mendel AI—Know More, Know Now. https://www.mendel.ai/.

[26] Medical-Chat Bot: The History of Our Attempt to Do It—Andrey Lukyanenko. https://andlukyane.com/blog/medical-chat-bot.

[27] Bouabida K., Lebouché B., Pomey M.-P. (2022). Telehealth and COVID-19 Pandemic: An Overview of the Telehealth Use, Advantages, Challenges, and Opportunities during COVID-19 Pandemic. Healthcare.

[28] Ahaskar A. How WhatsApp Chatbots Are Helping in the Fight against COVID-19. https://www.livemint.com/technology/tech-news/how-whatsapp-chatbots-are-helping-in-the-fight-against-covid-19-11585310168911.html.

[29] India’s Coronavirus Chatbot on WhatsApp Crosses 1.7 Crore Users in 10 Days. https://www.gadgets360.com/apps/news/coronavirus-mygov-corona-helpdesk-chatbot-whatsapp-indian-government-total-users-haptik-2204458.

[30] Matic R., Kabiljo M., Zivkovic M., Cabarkapa M. (2021). Extensible Chatbot Architecture Using Metamodels of Natural Language Understanding. Electronics.

[31] Developing AI Chatbots: Challenges and Considerations. https://www.linkedin.com/pulse/developing-ai-chatbots-challenges-considerations-artemakis-artemiou-yelif.

[32] Hien H.T., Cuong P.-N., Nam L.N.H., Nhung H.L.T.K., Thang L.D. (2018). Intelligent Assistants in Higher-Education Environments: The FIT-EBot, a Chatbot for Administrative and Learning Support. Proceedings of the 9th International Symposium on Information and Communication Technology.

[33] Kucherbaev P., Bozzon A., Houben G.-J. (2018). Human-Aided Bots. IEEE Internet Comput..

[34] Ta V., Griffith C., Boatfield C., Wang X., Civitello M., Bader H., DeCero E., Loggarakis A. (2020). User Experiences of Social Support From Companion Chatbots in Everyday Contexts: Thematic Analysis. J. Med. Internet Res..

[35] Medeiros L., Gerritsen C., Bosse T., Nguyen N.T., Chbeir R., Exposito E., Aniorté P., Trawiński B. (2019). Towards Humanlike Chatbots Helping Users Cope with Stressful Situations. Proceedings of the Computational Collective Intelligence.

[36] Medeiros L., Bosse T., Gerritsen C. (2022). Can a Chatbot Comfort Humans? Studying the Impact of a Supportive Chatbot on Users’ Self-Perceived Stress. IEEE Trans. Hum. Mach. Syst..

[37] Jin E., Eastin M.S. (2022). Birds of a Feather Flock Together: Matched Personality Effects of Product Recommendation Chatbots and Users. J. Res. Interact. Mark..

[38] Badlani S., Aditya T., Dave M., Chaudhari S. Multilingual Healthcare Chatbot Using Machine Learning. Proceedings of the 2021 2nd International Conference for Emerging Technology (INCET).

[39] Rojc M., Ariöz U., Šafran V., Mlakar I. (2023). Multilingual Chatbots to Collect Patient-Reported Outcomes. Chatbots—The AI-Driven Front-Line Services for Customers.

[40] Darius Z., Hundertmark S. (2017). Chatbots—An interactive technology for personalized communication, transactions and services. IADIS Int. J. WWW/Internet.

[41] Reshmi S., Balakrishnan K. (2018). Empowering chatbots with business intelligence by big data integration. Int. J. Adv. Res. Comput. Sci..

[42] Hopkins A.M., Logan J.M., Kichenadasse G., Sorich M.J. (2023). Artificial Intelligence Chatbots Will Revolutionize How Cancer Patients Access Information: ChatGPT Represents a Paradigm-Shift. JNCI Cancer Spectr..

[43] Sheehan B., Jin H.S., Gottlieb U. (2020). Customer Service Chatbots: Anthropomorphism and Adoption. J. Bus. Res..

[44] Følstad A., Skjuve M., Brandtzaeg P.B., Bodrunova S.S., Koltsova O., Følstad A., Halpin H., Kolozaridi P., Yuldashev L., Smoliarova A., Niedermayer H. (2019). Different Chatbots for Different Purposes: Towards a Typology of Chatbots to Understand Interaction Design. Proceedings of the Internet Science.

[45] Sharma P. (2023). Chatbots in Medical Research: Advantages and Limitations of Artificial Intelligence–Enabled Writing With a Focus on ChatGPT as an Author. Clin. Nucl. Med..

[46] Clark M., Bailey S. (2024). Chatbots in Health Care: Connecting Patients to Information. Can. J. Health Technol..

[47] Fan X., Chao D., Zhang Z., Wang D., Li X., Tian F. (2021). Utilization of Self-Diagnosis Health Chatbots in Real-World Settings: Case Study. J. Med. Internet Res..

[48] Sadasivan C., Cruz C., Dolgoy N., Hyde A., Campbell S., McNeely M., Stroulia E., Tandon P. (2023). Examining Patient Engagement in Chatbot Development Approaches for Healthy Lifestyle and Mental Wellness Interventions: Scoping Review. J. Particip. Med..

[49] Kowatsch T., Nißen M., Shih C.-H.I., Rüegger D., Volland D., Filler A., Künzler F., Barata F., Hung S., Büchter D. (2017). Text-Based Healthcare Chatbots Supporting Patient and Health Professional Teams: Preliminary Results of a Randomized Controlled Trial on Childhood Obesity.

[50] Rana J. (2023). The Pros and Cons of Healthcare Chatbots.

[51] Chen D., Parsa R., Hope A., Hannon B., Mak E., Eng L., Liu F.-F., Fallah-Rad N., Heesters A.M., Raman S. (2024). Physician and Artificial Intelligence Chatbot Responses to Cancer Questions from Social Media. JAMA Oncol..

[52] Wang A., Qian Z., Briggs L., Cole A.P., Reis L.O., Trinh Q.-D. (2023). The Use of Chatbots in Oncological Care: A Narrative Review. Int. J. Gen. Med..

[53] Tawfik E., Ghallab E., Moustafa A. (2023). A Nurse versus a Chatbot—The Effect of an Empowerment Program on Chemotherapy-Related Side Effects and the Self-Care Behaviors of Women Living with Breast Cancer: A Randomized Controlled Trial. BMC Nurs..

[54] Bibault J.-E., Chaix B., Guillemassé A., Cousin S., Escande A., Perrin M., Pienkowski A., Delamon G., Nectoux P., Brouard B. (2019). A Chatbot Versus Physicians to Provide Information for Patients With Breast Cancer: Blind, Randomized Controlled Noninferiority Trial. J. Med. Internet Res..

[55] Melián-González S., Gutiérrez-Taño D., Bulchand-Gidumal J. (2021). Predicting the Intentions to Use Chatbots for Travel and Tourism. Curr. Issues Tour..

[56] Okonkwo C.W., Ade-Ibijola A. (2021). Chatbots Applications in Education: A Systematic Review. Comput. Educ. Artif. Intell..

[57] Ozuem W., Ranfagni S., Willis M., Salvietti G., Howell K. (2024). Exploring the Relationship between Chatbots, Service Failure Recovery and Customer Loyalty: A Frustration–Aggression Perspective. Psychol. Mark..

[58] Grudin J., Jacques R. Chatbots, Humbots, and the Quest for Artificial General Intelligence. Proceedings of the 2019 CHI Conference on Human Factors in Computing Systems.

[59] Gupta M. Chatbots—Boon or Bane?. https://blog.bluelupin.com/chatbot-advantages-and-disadvantages/.

[60] Caldarini G., Jaf S., McGarry K. (2022). A Literature Survey of Recent Advances in Chatbots. Information.

[61] Yang J., Chen Y.-L., Por L.Y., Ku C.S. (2023). A Systematic Literature Review of Information Security in Chatbots. Appl. Sci..

[62] Bang J., Kim S., Nam J.W., Yang D.-G. Ethical Chatbot Design for Reducing Negative Effects of Biased Data and Unethical Conversations. Proceedings of the 2021 International Conference on Platform Technology and Service (PlatCon).

[63] Singh J., Sillerud B., Singh A. (2023). Artificial Intelligence, Chatbots and ChatGPT in Healthcare—Narrative Review of Historical Evolution, Current Application, and Change Management Approach to Increase Adoption. J. Med. Artif. Intell..

[64] Why Is AI Adoption in Health Care Lagging?. https://www.brookings.edu/articles/why-is-ai-adoption-in-health-care-lagging/.

[65] Brown J.E.H., Halpern J. (2021). AI Chatbots Cannot Replace Human Interactions in the Pursuit of More Inclusive Mental Healthcare. SSM Ment. Health.

[66] Xiao Z., Liao Q.V., Zhou M., Grandison T., Li Y. (2023). Powering an AI Chatbot with Expert Sourcing to Support Credible Health Information Access. Proceedings of the 28th International Conference on Intelligent User Interfaces.

[67] Tuncel F., Mumcu B., Tanberk S. A Chatbot for Preliminary Patient Guidance System. Proceedings of the 2021 29th Signal Processing and Communications Applications Conference (SIU).

[68] Kuroiwa T., Sarcon A., Ibara T., Yamada E., Yamamoto A., Tsukamoto K., Fujita K. (2023). The Potential of ChatGPT as a Self-Diagnostic Tool in Common Orthopedic Diseases: Exploratory Study. J. Med. Internet Res..

[69] Chen S., Kann B.H., Foote M.B., Aerts H.J.W.L., Savova G.K., Mak R.H., Bitterman D.S. (2023). Use of Artificial Intelligence Chatbots for Cancer Treatment Information. JAMA Oncol..

[70] Pan A., Musheyev D., Bockelman D., Loeb S., Kabarriti A.E. (2023). Assessment of Artificial Intelligence Chatbot Responses to Top Searched Queries About Cancer. JAMA Oncol..

[71] Tantsiura P. 5 Challenges of Chatbots for Business and How to Overcome Them. https://theappsolutions.com/blog/development/challenges-of-chatbots-for-business/.

[72] Fritsch T., Arai K. (2024). Chatbots: An Overview of Current Issues and Challenges. Proceedings of the Advances in Information and Communication.

[73] Loh E. (2024). ChatGPT and Generative AI Chatbots: Challenges and Opportunities for Science, Medicine and Medical Leaders. BMJ Lead..

[74] Gökçearslan Ş., Tosun C., Erdemir Z.G. (2024). Benefits, Challenges, and Methods of Artificial Intelligence (AI) Chatbots in Education: A Systematic Literature Review. IJTE.

[75] Tinmaz H., Lee Y.-T., Fanea-Ivanovici M., Baber H. (2022). A Systematic Review on Digital Literacy. Smart Learn. Environ..

[76] Rezaeikhonakdar D. (2024). AI Chatbots and Challenges of HIPAA Compliance for AI Developers and Vendors. J. Law Med. Ethics.

[77] Fink J. (2023). Can Artificial Intelligence Chatbots Convincingly Mimic Empathy?. Am. J. Nurs..

[78] Altamimi I., Altamimi A., Alhumimidi A.S., Altamimi A., Temsah M.-H. (2023). Artificial Intelligence (AI) Chatbots in Medicine: A Supplement, Not a Substitute. Cureus.

[79] Overman T., Blum G., Klabjan D. (2022). A Primal-Dual Algorithm for Hybrid Federated Learning. arXiv.

[80] Benjamin R. (2019). Race after Technology: Abolitionist Tools for the New Jim Code.

[81] Cath C. (2018). Governing Artificial Intelligence: Ethical, Legal and Technical Opportunities and Challenges. Phil. Trans. R. Soc. A.

[82] Cohen I.G., Amarasingham R., Shah A., Xie B., Lo B. (2014). The Legal And Ethical Concerns That Arise From Using Complex Predictive Analytics In Health Care. Health Aff..

[83] Vayena E., Blasimme A., Cohen I.G. (2018). Machine Learning in Medicine: Addressing Ethical Challenges. PLoS Med..

[84] Almalki M., Azeez F. (2020). Health Chatbots for Fighting COVID-19: A Scoping Review. Acta Inf. Med..

[85] Schillaci C.E., de Cosmo L.M., Piper L., Nicotra M., Guido G. (2024). Anthropomorphic Chatbots’ for Future Healthcare Services: Effects of Personality, Gender, and Roles on Source Credibility, User Satisfaction, and Intention to Use. Technol. Forecast. Soc. Change.

[86] Suppadungsuk S., Thongprayoon C., Miao J., Krisanapan P., Qureshi F., Kashani K., Cheungpasitporn W. (2023). Exploring the Potential of Chatbots in Critical Care Nephrology. Medicines.

[87] Palanica A., Flaschner P., Thommandram A., Li M., Fossat Y. (2019). Physicians’ Perceptions of Chatbots in Health Care: Cross-Sectional Web-Based Survey. J. Med. Internet Res..

[88] Tripathi S., Tabari A., Mansur A., Dabbara H., Bridge C.P., Daye D. (2024). From Machine Learning to Patient Outcomes: A Comprehensive Review of AI in Pancreatic Cancer. Diagnostics.

[89] Mouliou D.S. (2023). C-Reactive Protein: Pathophysiology, Diagnosis, False Test Results and a Novel Diagnostic Algorithm for Clinicians. Diseases.

[90] Mouliou D.S., Gourgoulianis K.I. (2021). False-Positive and False-Negative COVID-19 Cases: Respiratory Prevention and Management Strategies, Vaccination, and Further Perspectives. Expert. Rev. Respir. Med..

[91] Mouliou D.S., Dardiotis E. (2022). Current Evidence in SARS-CoV-2 mRNA Vaccines and Post-Vaccination Adverse Reports: Knowns and Unknowns. Diagnostics.

[92] Mouliou D.S., Gourgoulianis K.I. (2022). COVID-19 ‘Asymptomatic’ Patients: An Old Wives’ Tale. Expert. Rev. Respir. Med..

[93] Mouliou D.S., Pantazopoulos I., Gourgoulianis K. (2022). COVID-19 Diagnosis in the Emergency Department: Seeing the Tree but Losing the Forest. Emerg. Med. J..

[94] Mouliou D.S. (2023). The Deceptive COVID-19: Lessons from Common Molecular Diagnostics and a Novel Plan for the Prevention of the Next Pandemic. Diseases.

[95] Mouliou D.S. (2022). Managing Viral Emerging Infectious Diseases via Current Molecular Diagnostics in the Emergency Department: The Tricky Cases. Expert Review of Anti-infective Therapy. Expert Rev. Anti-Infect. Ther..

[96] Mouliou D.S. (2024). John Cunningham Virus and Progressive Multifocal Leukoencephalopathy: A Falsely Played Diagnosis. Diseases.

